# An Algorithm for Fitting Sphere Target of Terrestrial LiDAR

**DOI:** 10.3390/s21227546

**Published:** 2021-11-13

**Authors:** Yintao Shi, Gang Zhao, Maomei Wang, Yi Xu, Dadong Zhu

**Affiliations:** 1 School of Geomatics Science and Technology, Nanjing Tech University, Nanjing 211816, China; 2 Jiangsu Hydraulic Research Institute, Nanjing 210017, China; wangmaomei@163.com (M.W.); xuyi@163.com (Y.X.); zhudadong@163.com (D.Z.)

**Keywords:** light detection and ranging, point cloud, sphere target, constraint space, parameters, robustness

## Abstract

The sphere target played a vital role in terrestrial LiDAR applications, and solving its geometrical center based on point cloud was a widely concerned problem. In this study, we proposed a newly finite random search algorithm for sphere target fitting. Based on the point cloud data and the geometric characteristics of the sphere target, the algorithm realized the target sphere fitting from the perspective of probability and statistics with the help of parameter estimation. Firstly, an initial constraint space was constructed, and the initial center and radius were determined by finite random search. Then, the optimal spherical center and radius were determined gradually through continuous iterative optimization. We tested the algorithm with the simulated and realistic point cloud. Experimental results showed that the proposed algorithm could be effectively applied to all kinds of point cloud fitting. When the coverage rate was bigger than 30%, the fitting accuracy could reach within 0.01 mm for all kinds of point clouds. When the coverage rate was less than 20%, the fitting accuracy can reach ±1 mm, although it was reduced to a certain extent.

## 1. Introduction

Terrestrial light detection and ranging (LiDAR), also known as terrestrial laser scanning (TLS), could quickly acquire the high-resolution point cloud on the target surface by high-speed laser scanning and had brought the traditional single point measurement into the era of surface measurement. A point cloud was a set of data points in space, which was a collection of a large number of discrete measuring points on the external surface of an object. Each point position had its set of Cartesian coordinates (*X*, *Y*, *Z*). TLS technology had the characteristics of active, non-contact, high resolution, high precision, and rapid and flexible data acquisition. It could go deep into the complex field environment and realize the complete collection of the various large, irregular and non-standard entity or real scene 3D data. It has been wildly used in many works, such as engineering surveys, cultural relic protection, disaster monitoring, reverse 3D reconstruction et al. [1,2,3,4,5]. The sphere target (ST) had a typical spatial rotation symmetry and standard parameterized form. Part of its contour information could be obtained from any angle of view, with which the spherical center and radius could be effectively solved. Therefore, it was widely used in the multi-class application research of terrestrial LiDAR, such as the calibration and check of a terrestrial laser scanner, scanning accuracy evaluation, registration, and georeferencing of point clouds et al. [6,7,8,9,10,11]. The geometric center of the sphere target was inside the sphere and could not be obtained directly through measurement. Usually, what we utilized TLS to collect directly was the point cloud data on the surface of the target ball. Based on such data to determine its internal geometric center, this involved the fitting problem of the sphere target. At the same time, the sphere fitting problem was also a common problem to be solved in object tracking, pattern recognition, robotics, camera calibration, and other research work [12,13,14,15,16,17].

Judging from the related literature, some scholars have conducted related research on the problem of sphere fitting. Forbes took the center and the radius of the sphere as the parameters to be sought and analyzed the fitting algorithms of several types of spheres and other geometric bodies. These algorithms were mainly suitable for noise-free point cloud data with a high coverage rate (CR) [18]. Nievergelt used a least-squares method based on algebraic distances to calculate the center of the sphere. Although his method had advantages in computational efficiency, it usually did not provide satisfactory results [19]. Späth, Shakarji, and Ahn, et al. used improved least-squares methods to perform sphere fitting [20,21,22,23]. Clouse used conjugate gradient descent to calculate the sphere’s center, which used both cost function evaluations, and evaluations of the derivative to find a set of parameters that produce a local minimum cost [24]. Witzgall respectively used algebraic fitting and geometric fitting to perform sphere fitting. With the help of the concept of deviation between data point and sphere, the arithmetic fitting was solved by least-square through linear regression. The geometric fitting used the orthogonal least-squares solution [25]. Sumith used a fast geometric method to fit the center and radius of the sphere, and the fitting accuracy was better than the ordinary least squares estimator (OLS) [26]. Liu used a nonlinear least-squares method to achieve sphere fitting [27]. Fei used a constrained nonlinear least-squares fitting (CNLSF) algorithm to realize the fitting of spheres with a small segment angles strategy [28]. Lesouple used an expectation-maximization method to achieve the fitting of spheres [29].

At present, most of the sphere fitting algorithms mainly rely on least-squares minimization methods to obtain their centers, such as linear least-squares, nonlinear least-squares, the total least squares method as well as the weighted total least squares method to eliminate the error of the coefficient matrix [30,31,32]. From the theory of least squares, the least-squares estimation assumed that the mean of data noise was zero, resulting in an unbiased parameter estimation. If the noise variance was known, the minimum variance parameter estimation could be obtained by selecting appropriate weights on the data. In addition, least squares estimation implicitly assumed that the entire data set could only be explained by one parameter vector of a given model [33,34]. Numerous studies have clearly shown that least-squares estimation could easily violate these assumptions. Sometimes, even if the data contained only one “bad“ datum, the least-squares estimate may be seriously disturbed, resulting in low calculation accuracy. In addition to the least-squares method, there were also some other methods, such as a minimum zone sphere, maximum inscribed sphere, minimum circumscribed sphere [35,36,37]. These methods mainly take advantage of linearization to fit the sphere with the help of mathematics or geometry. The sphere target fitting itself was a nonlinear problem, which inevitably led to the loss of accuracy in the linearization process. At the same time, the number of points in a sphere target point cloud was usually more than thousands, which would cause a large calculation matrix and low computational efficiency.

As we all know, in TLS work, no matter what type of sphere target we used, it had a specific geometric size, that is to say, the spatial distribution of the point cloud of any sphere target had a particular range that we call bounding box, and this bounding box contains the whole point cloud of the sphere target, including the noises. From the geometric characteristics, the geometric center and radius of the sphere target must be in this bounding box. Thus, we could adopt a search strategy to look for the optical center and radius in the bounding box that satisfies the specific error criteria. In this study, combining the point cloud and geometric characteristics of point cloud, we developed a finite random search alogorithm for the sphere target fitting. Our proposed algorithm mainly aimed to achieve a better sphere target fitting after the point cloud extraction of a singular sphere target has been completed. Its main objective is to calculate the geometric center accurately based on the point cloud data of a single target sphere. The detailed design of the algorithm is described in Section 2. In this paper, we did not discuss how to extract point cloud data of an individual target sphere from a complex point cloud, but there were many solutions for this problem [38].

## 2. Methods and Data

Given a point cloud of a sphere target $T=\left\{\left(x_{i},y_{i},z_{i}\right)|i=1,2,\cdots ,n\geq 3\right\}$ obtained by TLS, let (*X*, *Y*, *Z*) be the unknown center, and let R be the unknown radius of the sphere target. In a specific scanning coordinate system, both the geometric center (*X*, *Y*, *Z*) and radius R of the sphere target were determined. During the data acquisition process, affected by factors such as the instrument itself and the external environment, a point cloud was inevitably mixed with noise [39,40]. Sphere target fitting was to extract the center and radius of the sphere target from the point cloud with unknown distribution and outliers. This could be viewed as an optimal parameter estimation problem. In this problem, we regarded the geometric center (*X*, *Y*, *Z*) and radius R of the sphere target as the parameters to be solved and took the target point cloud as the observation value. Using the point cloud to fit the geometric center and radius could be regarded as finding the optimal parameters that meet the specific decision rules.

**Figure 1:**
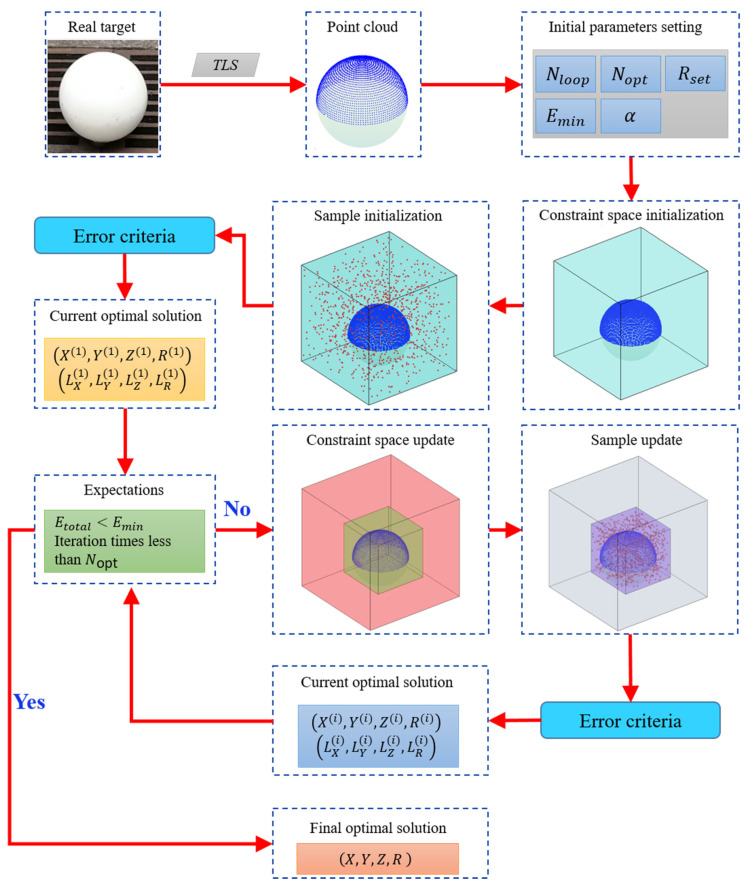
Technical flow of our algorithm.

We took the centroid of the sphere target point cloud as the center and took more than two times the radius length as the constraint to construct an initial bounding box. According to the geometric characteristics of the sphere target, its geometric center and radius must be within the bounding box. Based on this feature, we could solve the problem of the sphere target fitting by using the idea of probability theory and parameter estimation. Let each sample in sample space $U=\left\{\left(X_{i},Y_{i},Z_{i},R_{i}\right)|i=1,2,\cdots ,n\right\}$ be composed of four characteristic quantities, where $\left(X_{i},Y_{i},Z_{i}\right)$ was the potential geometric center of the target sphere and $R_{i}$ was the potential geometrical radius of the target sphere. The four characteristic quantities (*X*, *Y*, *Z*, *R*) were continuous variables, and their values should be infinite in theory. From the perspective of probability and statistics, in the process of finite random search, the probability of obtaining the optimal value was related to the size of the sample space. The larger the sample space, the lower the probability of finding the optimal value. Conversely, the smaller the sample space, the higher the probability of finding the optimal value [41,42]. In this study, we proposed a finite random search algorithm suitable for sphere target fitting combined with the point cloud and geometric characteristics of the sphere target. The primary technical process of the algorithm is shown in Figure 1.

### 2.1. Initial Parameters

The initial parameters were mainly composed of the random search times $N_{\mathrm{loop}}$, the iterative optimization times $N_{\mathrm{opt}}$, the estimated radius of the target sphere $R_{\mathrm{set}}$, the total error threshold $E_{min}$ and the scale scaling factor α.

- $N_{\mathrm{loop}}$ referred to the number of random searches for the optimal value in a set-limited search space.
- $N_{\mathrm{opt}}$ referred to the predetermined number of times to update the search space in the iterative search process.
- $R_{\mathrm{set}}$ referred to the pre-estimated geometric radius of the sphere target. In TLS work, the geometric radius of the sphere target used was usually known, and if the radius could not be determined, $R_{\mathrm{set}}$ could be set to a relatively sizeable rough value.
- $E_{min}$ referred to the preset value of the total error that determined the end of the fitting prematurely. Setting the threshold here could realize that after the predetermined fitting accuracy was reached, the fitting process was ended early to improve the execution efficiency of the algorithm.
- α referred to the adjustment factor of the constrained space scaling when the constrained space was updated.

### 2.2. Error Criteria

The fitting problem of the sphere target point cloud could be regarded as the problem of estimating parameters from noisy data, where the judgment criterion played an important role. That would influence the accuracy of the estimated parameters, computation efficiency, and the robustness to predictable or unpredictable errors. To find the optimal center and radius of the target sphere, we must first determine the optimal center and radius measurement criteria. The error criteria of the existing sphere target fitting algorithm were mainly divided into geometric error and arithmetic error, each of which has its advantages and disadvantages [43]. In our proposed algorithm, an arithmetic error was chosen as the criterion.

Let the current fitting center of the target sphere be (X,Y,Z) and the radius *R*. The current total error $E_{total}$ could be calculated by Equation (1).

(1) $$E_{total}=\sum_{i=1}^{n}\left|{\left(x_{i}-X\right)}^{2}+{\left(y_{i}-Y\right)}^{2}+{\left(z_{i}-Z\right)}^{2}-R^{2}\right|$$

where $\left(x_{i},y_{i},z_{i}\right)$ were the coordinates of the measured points of the point cloud, and *n* was the number of the measuring points contained in the point cloud.

In the proposed algorithm, a limited sample of possible solutions was generated with the help of the constraint space, and their total error was calculated by using Equation (1) one by one. The sample with the smallest total error was taken as the optimal solution under the current constraint space. More detailed usage is described in Section 2.3 and Section 2.4. Theoretically, the smaller the total error $E_{total}$ was, the higher the coincidence degree between the point cloud and simulated sphere surface was, the more accurate the current center and radius were; otherwise, the deviation of the center or radius was more significant.

### 2.3. Initial Constraint Space and Sample Construction

**Figure 2:**
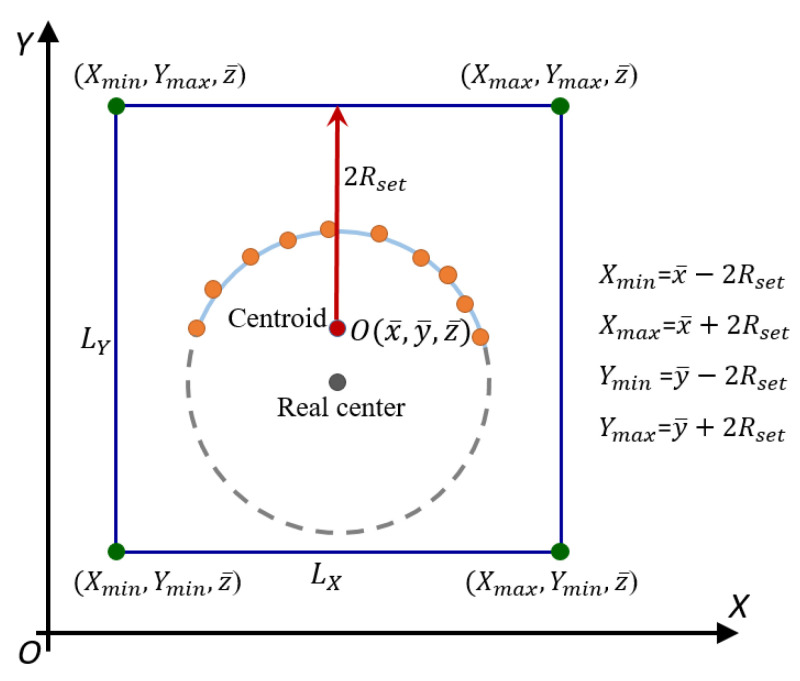
Schematic diagram of 2D initial space construction.

In terms of the geometric characteristics of the sphere target, given the point cloud of a sphere target, its centroid could be solved, and this centroid could be used as the geometric center of the bounding box of the point cloud. By adjusting the side length of the bounding box, we could construct a space cube containing the point cloud of the sphere target and the complete range of the sphere target to be fitted. Then, the center of the sphere target must be in this space cube. We called this cube the constrained space S, and the cube constructed for the first time was the initial constraint space $S^{\left(1\right)}$. In the algorithm in this paper, the initial constraint space was mainly constructed based on the point cloud and the preset parameter $R_{\mathrm{set}}$, and the construction principle is shown in Figure 2.

(1) The centroid $O\left(\bar{x},\bar{y},\bar{z}\right)$ of the sphere target point cloud could be calculated and used as the center of the initial constraint space.

(2) With *O* as the geometric center and $R_{set}$ as the constraint, the space ranges of the X, Y, and Z axes of the initial constraint space and radius range were determined by Equation (2). In general, $R_{set}$ was set to be slightly larger than the real radius of the sphere target to ensure that the constructed initial constraint space covered the entire target sphere. In practical scanning operations, the sphere target used usually had a clear geometric size, so the estimated radius of the sphere target should be easy to determine.

(2) $$\left\{ \begin{array}{l} X^{\left(1\right)}:=\left[X_{min}^{\left(1\right)},X_{max}^{\left(1\right)}\right]=\left[\bar{x}-2R_{set},\bar{x}+2R_{set}\right]\\ Y^{\left(1\right)}:=\left[Y_{min}^{\left(1\right)},Y_{max}^{\left(1\right)}\;\right]=\left[\bar{y}-2R_{set},\bar{y}+2R_{set}\right]\\ Z^{\left(1\right)}:=\left[Z_{min}^{\left(1\right)},\;Z_{max}^{\left(1\right)}\right]=\left[\bar{z}-2R_{set},\bar{z}+2R_{set}\right]\\ R^{\left(1\right)}:=\left[R_{min}^{\left(1\right)},\;R_{max}^{\left(1\right)}\right]=[0,\;2R_{set}]\; \end{array}\right.$$

(3) With the initial constraint space and radius as constraints, a sample space $U^{\left(1\right)}=\left\{\left(X_{i}^{\left(1\right)},Y_{i}^{\left(1\right)},Z_{i}^{\left(1\right)},R_{i}^{\left(1\right)}\right)|i=1,2,\cdots ,N_{loop}\right\}$ composed of four characteristic quantities was constructed, and it contained $N_{loop}$ randomly generated samples.

(4)After determining the value ranges of the four characteristic quantities in the sample space, the current scale of the characteristic quantities $\left(L_{X}^{\left(1\right)},L_{Y}^{\left(1\right)},L_{Z}^{\left(1\right)},L_{R}^{\left(1\right)}\right)$ could be determined by Equation (3).

(3) $$\{\begin{array}{c} L_{X}^{\left(1\right)}=\left|X_{max}^{\left(1\right)}-X_{min}^{\left(1\right)}\right|\\ L_{Y}^{\left(1\right)}=\left|Y_{max}^{\left(1\right)}-Y_{min}^{\left(1\right)}\right|\\ L_{Z}^{\left(1\right)}=\left|Z_{max}^{\left(1\right)}-Z_{min}^{\left(1\right)}\right|\\ L_{R}^{\left(1\right)}=\left|R_{max}^{\left(1\right)}-R_{min}^{\left(1\right)}\right| \end{array}$$

The $E_{total}$ of each sample in $U^{\left(1\right)}$ was calculated in sequence by the error criterion of Section 2.2, and compared with the preset total error threshold $E_{min}$. If $E_{total}$ was less than $E_{min}$, it indicated that the current sample had reached the predetermined accuracy of parameter estimation. At this point, this sample could be considered as the optimal solution of parameters. Otherwise, the search should continue until all samples in $U^{\left(1\right)}$ were tested. After traversing all the samples in $U^{\left(1\right)}$, if no sample satisfying $E_{total}<E_{min}$ was found, the sample with the smallest $E_{total}$ was selected as the optimal solution of the parameter in the current constraint space.

### 2.4. Constraint Space and Sample Update

From the perspective of probability and statistics, in the process of finite random search, the probability of obtaining the optimal value was related to the size of the constraint space. The smaller the constraint space, the higher the probability of finding the optimal value [44,45]. In the last constraint space, the center and radius determined by finite random search did not meet the set accuracy, so the sample space needed to be further optimized to accurately determine the center and radius.

Suppose that in the constraint space $S^{\left(i\right)}$, the optimal solution of center and radius obtained from $N_{loop}$ samples was $\left(X^{\left(i\right)},Y^{\left(i\right)},Z^{\left(i\right)},R^{\left(i\right)}\right)$, and the scale of the characteristic quantity was $\left(L_{X}^{\left(i\right)},L_{Y}^{\left(i\right)},L_{Z}^{\left(i\right)},L_{R}^{\left(i\right)}\right)$.

(1) Each feature quantity could be scaled by using Equation (4). Here α∈(0,1) was the preset scale scaling factor, which was used to adjust the scaling speed of each feature quantity. The smaller the value was, the faster the scaling speed was.

(4) $$\{\begin{array}{c} L_{X}^{\left(i+1\right)}=\alpha L_{X}^{\left(i\right)}\\ L_{Y}^{\left(i+1\right)}=\alpha L_{Y}^{\left(i\right)}\\ L_{Z}^{\left(i+1\right)}=\alpha L_{Z}^{\left(i\right)}\\ L_{R}^{\left(i+1\right)}=\alpha L_{R}^{\left(i\right)} \end{array}$$

(2) With $\left(X^{\left(i\right)},Y^{\left(i\right)},Z^{\left(i\right)},R^{\left(i\right)}\right)$ as the center and the updated feature quantity scale $\left(L_{X}^{\left(i+1\right)},L_{Y}^{\left(i+1\right)},L_{Z}^{\left(i+1\right)},L_{R}^{\left(i+1\right)}\right)$ as the constraint, a new constraint space $S^{\left(i+1\right)}$ was constructed. The value ranges of the four characteristic quantities of each sample in sample space $U^{\left(i+1\right)}=\left\{\left(X_{i}^{\left(i+1\right)},Y_{i}^{\left(i+1\right)},Z_{i}^{\left(i+1\right)},R_{i}^{\left(i+1\right)}\right)|i=1,2,\cdots ,N_{loop}\right\}$ were determined by Equation (5). In this case, the value of $R_{min}^{\left(i+1\right)}$ should be paid attention. The radius of the sphere target should not be negative, so $R_{min}^{\left(i+1\right)}$ must always be greater than 0.

(5) $$\left\{ \begin{array}{l} X^{\left(i+1\right)}:=\left[X_{min}^{\left(i+1\right)},X_{max}^{\left(i+1\right)}\right]=\left[X^{\left(i\right)}-L_{X}^{\left(i+1\right)},X^{\left(i\right)}+L_{X}^{\left(i+1\right)}\right]\\ Y^{\left(i+1\right)}:=\left[Y_{min}^{\left(i+1\right)},Y_{max}^{\left(i+1\right)}\right]=\left[Y^{\left(i\right)}-L_{Y}^{\left(i+1\right)},Y^{\left(i\right)}+L_{Y}^{\left(i+1\right)}\right]\\ Z^{\left(i+1\right)}:\left[Z_{min}^{\left(i+1\right)},Z_{max}^{\left(i+1\right)}\right]=\left[Z^{\left(i\right)}-L_{Z}^{\left(i+1\right)},Z^{\left(i\right)}+L_{Z}^{\left(i+1\right)}\right]\\ R^{\left(i+1\right)}:=\left[R_{min}^{\left(i+1\right)},R_{max}^{\left(i+1\right)}\right]=\left[R^{\left(i\right)}-L_{R}^{\left(i+1\right)},R^{\left(i\right)}+L_{R}^{\left(i+1\right)}\right] \end{array}\right.$$

(3) With the updated constraint space $S^{\left(i+1\right)}$ and radius as constraints, a new sample space $U^{\left(i+1\right)}$ containing $N_{loop}$ samples was randomly generated.

According to the same search strategy, the $E_{total}$ of each sample in the sample space $U^{\left(i+1\right)}$ was calculated one by one and detected whether it was less than the preset threshold $E_{min}$ of the total error. After traversing all the samples in $U^{\left(i+1\right)}$, if no sample satisfying $E_{total}<E_{min}$ was found, the sample with the minor total error was selected as the optimal solution of the parameters in the current constraint space.

### 2.5. Iterative Optimization

Based on the constraint space constructed, the finite random sample space was generated, and the optimal sample in the sample space was determined as the optimal solution in the current constraint space. The optimal solution satisfying the predetermined accuracy was gradually found by constantly updating the search and sample spaces. In general, after more than ten iterations of optimization, the optimal value could be determined. In the iterative optimization process, the search process may end when $E_{total}$ was less than $E_{min}$, or it may end after the predetermined $N_{opt}$ iterative optimization. At the same time, considering the noise contained in the point cloud, the $E_{total}$ obtained may always fail to reach $E_{min}$ during the next finite iteration of $N_{opt}$, but after a finite iteration search, $E_{total}$ might always stop decreasing. At this time, it was necessary to detect a situation that if the $E_{total}$ did not decrease during two consecutive iterations, the iterative process should end.

### 2.6. Accuracy Estimation

The fitting accuracy of the sphere target center directly determined the scope of application of the algorithm. When the center of the sphere target was known, the fitting accuracy could be evaluated by the root mean square error (RMSE) of the center [46,47]. Let the real center of the sphere target be $\left(X_{o},Y_{o},Z_{o}\right)$ and the fitted center $\left(X_{f},Y_{f},Z_{f}\right)$. The RMSE of the fitted center could be obtained by Equation (6).

(6) $$RMSE=\pm \sqrt{\frac{{\left(X_{f}-X_{o}\right)}^{2}+{\left(Y_{f}-Y_{o}\right)}^{2}+{\left(Z_{f}-Z_{o}\right)}^{2}}{N}}\;=\pm \sqrt{\frac{dX^{2}+dY^{2}+dZ^{2}}{N}}$$

where in this study, *N =* 1. 

Because the center of the sphere target was located inside the sphere, it could not be measured directly, so it was difficult to accurately evaluate the fitting accuracy of the center of the sphere target in practical work. To effectively test the fitting effect of the proposed algorithm, simulation data with known center and radius were used to test the algorithm.

## 3. Experiment

To test the feasibility of the algorithm proposed in this paper, we designed two groups of simulated data without noise point cloud and noisy point cloud for testing. The noise-free point cloud mainly simulates the data processed by various denoising operations. The noisy point cloud mainly simulates the data obtained by TLS directly and without previous noise processing. The test platform uses Windows 10 system, Intel I5-6500 CPU, 16G memory, and Intel Graphics 530 Graphics card.

### 3.1. Point Cloud Simulation

**Figure 3:**
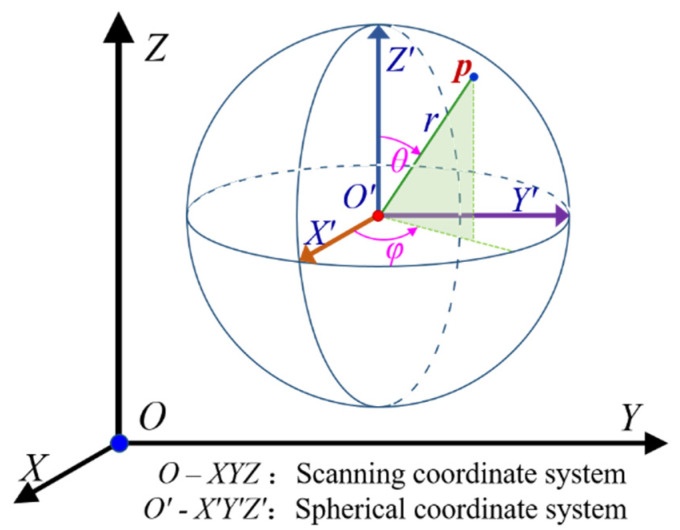
Schematic diagram of point cloud coordinates.

A target sphere was a standard spherical geometry whose point cloud consisted of several measuring points on the surface of the target sphere. The spherical coordinates (x,y,z) of any measurement point ***p*** in the sphere target point cloud could be determined by the spherical radius *r*, the zenith angle $\theta \in \left[0,180^{{}^{\circ}}\right]$ and the plane projection angle $\varphi \in \left[0,360^{{}^{\circ}}\right]$, as shown in Figure 3. In the scanning coordinate system, the scanning coordinate (*X*, *Y*, *Z*) of measuring point ***p*** was affected not only by spherical coordinates but also by the position of spherical coordinates $\left(x_{0},y_{0},z_{0}\right)$ in the scanning coordinate system. At this point, the scanning coordinate of measuring point ***p*** should be calculated by Equation (7). The sphere target point cloud was simulated by equally dividing the zenith angle *θ* and plane projection angle *φ*.

(7) $$\{\begin{array}{c} X=x_{0}+r\sin\theta \cos\varphi \\ Y=y_{0}+r\sin\theta \sin\varphi \\ Z=z_{0}+r\cos\theta \;\;\;\; \end{array}$$

**Figure 4:**
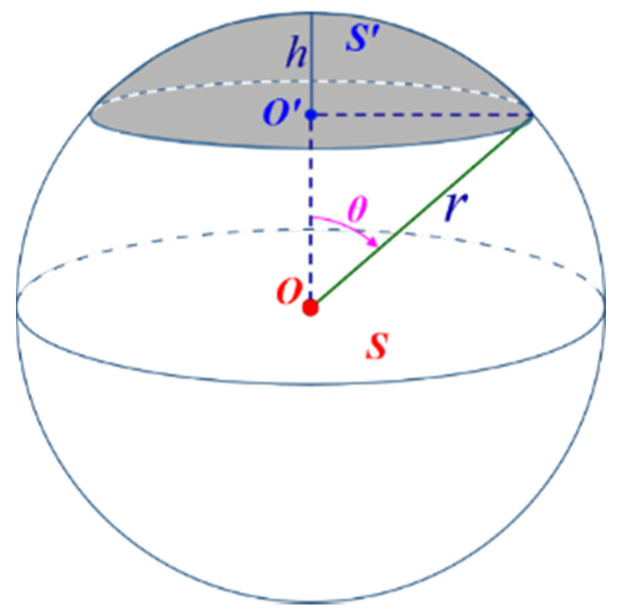
Schematic diagram of coverage rate of the point cloud.

Due to the influence of TLS scanning field of view, occlusion of the target sphere itself, scanning distance between TLS and sphere target, single station scanning could only obtain target sphere point cloud data with maximum coverage of 50% [48,49]. Coverage rate (CR) here was the percentage of the surface area covered by the pointing cloud to the total area of the target sphere. It was mainly calculated based on the proportion of the surface area *S*′ occupied by the point cloud to the sphere‘s surface area *S*, as shown in Figure 4.

The CR simulation of experimental data was realized by adjusting the range of zenith angle *θ*. According to the surface area calculation formula of the spherical crown and sphere, the coverage rate c of the simulated point cloud could be calculated by Equation (8), and the meanings of each parameter are shown in Figure 4 [50,51].

(8) $$c=\frac{S’}{S}=\frac{2\pi rh}{4\pi r^{2}}=\frac{h}{2r}$$

where *S*′ was the surface area of the spherical crown, *S* was the surface area of the sphere, *h* was the height of the spherical crown, and *r* was the radius of the sphere.

In the simulation of a sphere target point cloud, the coverage rate *c* of the point cloud was given, and the value range of the zenith angle $\theta \in \left(0,\theta_{\max}\right)$ should be determined according to the CR *c*, where $\theta_{max}$ could be calculated by Equation (9).

(9) $$\theta_{max}=\arccos\left(\frac{\bar{OO’}}{r}\right)=\arccos\left(\frac{r-h}{r}\right)=\arccos\left(1-2c\right)$$

where $\bar{OO’}$ was the distance from the sphere’s center to the underside of the spherical crown, and other symbols were the same as Equation (8).

Influenced by the instrument performance and external environment (wind, air humidity, illumination, etc.), noise would inevitably be mixed in the point cloud obtained by TLS. According to the error theory of geodesy, the noise in the measured data usually satisfied the Gaussian distribution $N\left(\mu ,\sigma^{2}\right)$, where *μ* and $\sigma^{2}$ were the expected and variance of the Gaussian distribution, respectively. In the simulation of experimental data, Gaussian noise was added to the point cloud to simulate the real noise in the scanning process. In all noise point cloud simulations, *μ* was set to 0, and *σ* was set to the maximum deviation value of the point cloud.

### 3.2. Noise-Free Point Cloud

**Figure 5:**
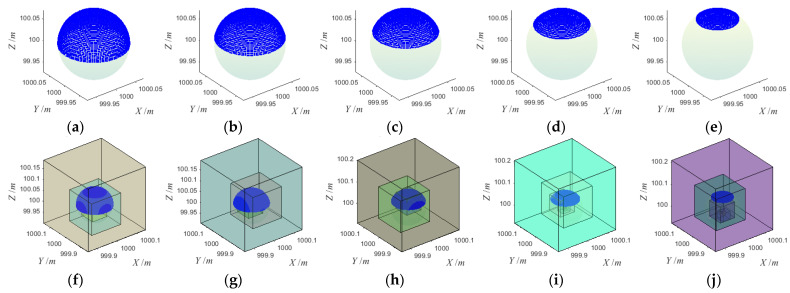
Simulation and fitting of noise-free point cloud: (**a**) CR 50%, (**b**) CR 40%, (**c**) CR 30%, (**d**) CR 20%, (**e**) CR 10, (**f**) fitting process of (**a**), (**g**) fitting process of (**b**), (**h**) fitting process of (**c**), (**i**) fitting process of (**d**), (**j**) fitting process of (**e**).

For noise-free point clouds of sphere target with different coverage rates, five simulated data were generated by using method 3.1, as shown in Figure 5. Among them, coverage rates of (a)~(e) were 50%, 40%, 30%, 20% and 10%, respectively. The center and radius (*X*, *Y*, *Z*, *R*) of all the simulated point clouds were (1000, 1000, 100, 0.0725), and the unit was the meter. The sampling interval of both the zenith angle *θ* and the plane projection angle *φ* was 3°. We use the proposed algorithm to fit the five simulated point clouds, and the fitting process is shown in Figure 5. For the initial parameter setting in the fitting process, $N_{\mathrm{loop}}$, $N_{\mathrm{opt}}$, $R_{\mathrm{set}}$, $E_{min}$, and *α* were 1000, 30, 0.08 m, 0.001, and 0.25, respectively.

After all sphere target fitting was completed, we calculated statistics on the number of points in the point cloud, the RMSE of the fitting center, the total error at the end of the fitting, iteration times, and running time, as shown in Table 1. According to the statistical results, for the noise-free point cloud, when the coverage rate reaches more than 30%, the total error threshold of 0.001 could be achieved, and the fitting center‘s RMSE was less than 0.01 mm after less than 15 iterations of optimization. When the coverage was below 20%, the iterative optimization times would increase, but generally, the fitting would be completed after about 25 iterations of optimization. At this time, the total error did not reach the predetermined total error threshold of 0.001, and fitting should be the end of the iteration process when the adjacent total error did not decrease, and both the deviations of X, Y, and Z axes and the fitting center‘s RMSE were all less than 1 mm.

Experimental results showed that the iterative optimization times were mainly affected by the coverage rate in the absence of noise. When the coverage rate was greater than 30%, ideal fitting results could be obtained after 15 iterations of optimization. When the coverage was less than 20%, the number of iterations would increase, but generally, the fitting could be completed after less than 25 iterations. The running time was mainly affected by the number of measuring points in the point cloud. When the coverage rate was high, the number of measuring points was large, and the running time was extended. When the coverage rate was low, the measuring point data was small, and the running time was short. From the calculation time of 10 simulated data, the fitting could be completed in less than 0.5 s.

### 3.3. Noisy Point Cloud

**Figure 6:**
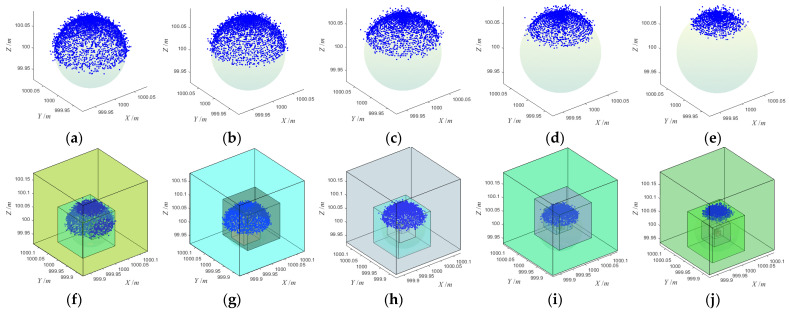
Simulation and fitting of noisy point cloud: (**a**) CR 50%, (**b**) CR 40%, (**c**) CR 30%, (**d**) CR 20%, (**e**) CR 10, (**f**) fitting process of (**a**), (**g**) fitting process of (**b**), (**h**) fitting process of (**c**), (**i**) fitting process of (**d**), (**j**) fitting process of (**e**).

Affected by instrument performance, scanning distance, incident angle, reflection intensity, surface roughness, the external environment (e.g., ambient vibration, wind, temperature), and other factors, the TLS point cloud cannot avoid mixed noise. From various scanners’ existing nominal technical parameters, the ranging error within the scanning distance of 100 m was basically within ±2.0 mm. Considering the influence of the surrounding environment and other factors, we add ±5.0 mm noise to the simulation data. We used method 3.1 to simulate five sphere target point clouds with different coverage rates of ±5 mm noise, as shown in Figure 6. Among them, the coverage rates of (a)~(e) were 50%, 40%, 30%, 20% and 10%, respectively. The center and radius (X, Y, Z, R) of all the simulated point clouds were (1000, 1000, 100, 0.0725), and the unit was the meter. The sampling interval of both the zenith angle *θ* and the plane projection angle *φ* was 3°. We use the proposed algorithm to fit the five simulated point clouds, respectively, and the fitting process is shown in Figure 6. The initial parameter setting of the algorithm was the same as that of the noise-free point cloud fitting, $N_{\mathrm{loop}}$, $N_{\mathrm{opt}}$, $R_{\mathrm{set}}$, $E_{min}$, and *α* were 1000, 30, 0.08 m, 0.001, and 0.25, respectively.

After every sphere target fitting was completed, we made statistics on the number of points in the point cloud, the RMSE of the fitting center, the total error at the end of the fitting, iteration times, and running time, as shown in Table 2. According to the statistical results, for the noisy point clouds with different coverage rates, the fitting process ends when the total errors stop decreasing during the iterative optimization process, and the total errors at the end of the process do not reach the preset total error threshold of 0.001. When the coverage was above 30%, the fitting center with RMSE less than 0.001 mm could be obtained after about 25 iterations of optimization. When the coverage rate was 20%, the fitting center with RMSE less than 1 mm could be obtained after about 20 iterations. When the coverage rate was 10%, the fitting center‘s RMSE was about 2 mm after about 20 iterations of optimization. At this time, the error of X, Y, and Z axes primarily occurs in the *Z*-axis, because the point cloud and noise were mainly concentrated in the Z-axis, which may affect the fitting accuracy of the sphere target center in this direction to some extent.

It could be seen from the comparison of the fitting results of noise-free point cloud and noisy point cloud when the point cloud was mixed with noise, the number of iterative optimization of point cloud with coverage of more than 30% increases obviously, while the number of iterative optimization of point cloud with coverage of less than 20% does not change significantly. The accuracy of the fitting center was mainly affected by the coverage rate. Whether there was noise mixed in the point cloud or not, the RMSE of the fitting center was less than 0.01 mm when the coverage rate reached more than 30%. The fitting accuracy of the point cloud with a 20% coverage rate was about 1 mm. The point cloud with a 10% coverage rate was susceptible to noise, and the fitting accuracy would be reduced to a certain extent after mixing with noise. The running time was mainly restricted by the number of measuring points in the point cloud, but noise and coverage rate was not significantly affected. The more the number of measuring points, the longer the runtime would be.

### 3.4. Realistic Point Cloud

**Figure 7:**
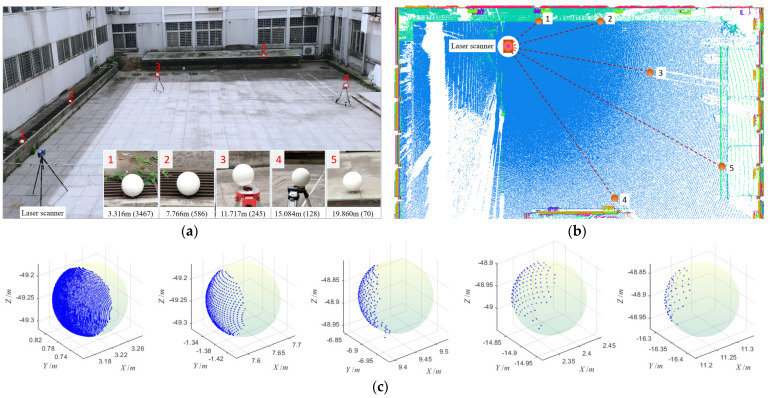
Acquisition of real sphere target point clouds. (**a**) Realistic experiment scene and sphere target arrangement. According to their distance from the scanner, they were numbered 1~5 from near to far, and their distances from the scanner and the numbers of measuring points contained in each point cloud were given. (**b**) Plane projection of the original point cloud of the whole scene. It clearly revealed the spatial distribution of the experimental sphere target. (**c**) The real point cloud of five sphere targets. The virtual spheres were used for auxiliary display.

In order to test the applicability of the proposed algorithm to real target balls, the point clouds of five real sphere targets were acquired by TLS. The experimental site was selected in a small square on the campus of Nanjing Tech University, where five sphere targets with different distances were arranged, and the Faro Laser Scanner Focus^3D^ X330 was used to collect their point clouds. The arrangement of the Target balls is shown in Figure 7a. According to their distance from the scanner, they were named Target 1~5 respectively from near to far. The sphere target selected was Faro’s standard sphere target, whose real geometric radius was 0.0725 m. The FARO Focus^3D^ X330 was a high-speed 3D scanner with extra-long range, which could scan objects up to 330 m away even in direct sunlight. Its nominal ranging error was ±2 mm, and ranging noise was 0.3 mm at a distance of 10 and 25 m when the reflectivity was 90%. The point cloud of the whole scene was obtained through single-site scanning according to the point spacing setting of 7.67 mm@10 m, as shown in Figure 7b. According to the measurement principle of a 3D laser scanner, the spatial distribution of scanned point cloud showed divergence, that is, the farther away from the scanner, the larger the point spacing and the smaller the spatial density, which could be clearly shown in the orthographic projection of the scanned point cloud in Figure 7b. From the original point clouds obtained without any denoising process, point clouds of five sphere targets were manually extracted, as shown in Figure 7c.

In practical scanning work, the real geometric center of the sphere target could not be measured directly, so its true value cannot be accurately obtained. In order to effectively compare the fitted results of the proposed algorithm with other software or algorithms, the geometric centers of five target spheres were extracted by the point cloud processing software of Faro’s SCENE and regarded as reference values. In SCENE software, the fitting of the sphere target needed to set the radius of the sphere target in advance, which was set as the real radius of the target ball (0.0725 m). Meanwhile, our algorithm and the least square (LS) algorithm were used to fit the five point clouds, respectively, and the fitting results were compared with the reference values, as shown in Table 3. Here, dX, dY, dZ and dR were the absolute values of deviations between the fitted center and radius of the sphere target and the reference value, respectively, and RMSE was the error value of the fitted center, which could be calculated by Equation (5). The preset parameters required in our algorithm were the same as those in Section 3.2. When LS was applied to fit, the initial values of center and radius needed to be given, which were respectively set as the centroid of the point cloud and the real radius of the sphere target.

From the scanning and fitting experiments of real sphere targets, the following conclusions could be drawn. 

**Figure 8:**
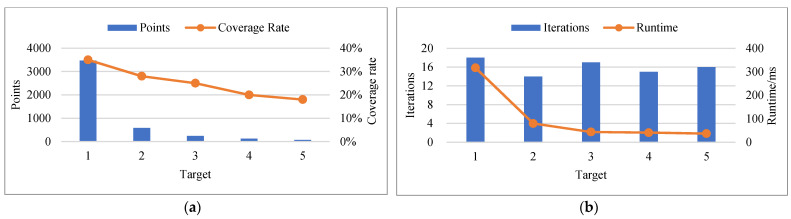

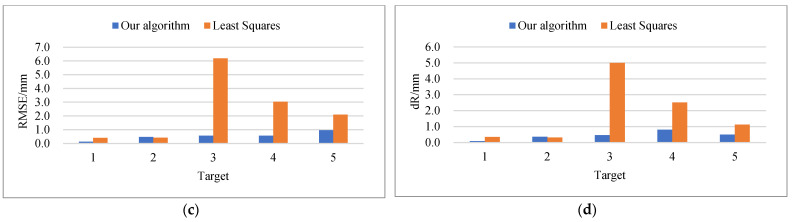
Analysis of experimental results of real sphere targets. (**a**) The relationship between the scanning distances and the points and coverage rates of the point clouds. (**b**) Statistics of iteration optimization times and runtime of our algorithm. (**c**) Comparison of RMSE of fitted centers between our algorithm and LS algorithm. (**d**) Comparison of the error of fitted radius between our algorithm and LS algorithm.

(1) The points and coverage rate of the point cloud were directly affected by the distance between the sphere target and the scanner. It could be seen from Figure 8a that as the distance between the sphere target and the scanner increased, both the number of measuring points and the coverage rate decreased accordingly, which was determined by the performance of the instrument. In actual scanning work, the coverage rate was usually less than 40%. For example, Target 1, which was 3.316 m away from the scanner, had a coverage rate of only 35%.

(2) Our algorithm was efficient in real sphere target fitting. From the iterative optimization times and runtime, our algorithm could complete the fitting after less than 20 iterative optimizations, and the runtime was less than 0.5 s, as shown in Figure 8b. 

(3) The fitting accuracy of our algorithm was comparable to that of commercial software SCENE. Under the assumption that the centers of the sphere targets by SCENE were the true value, the deviation of X, Y and Z and RMSE of the fitted center of our algorithm were all less than 1 mm, as shown in Table 3. From another perspective, the applicability of our algorithm was better than that of commercial software SCENE. The reason was that in SCENE’s fitting work, the true radius of the sphere target must first be accurately set, but our algorithm only needed a rough estimate, and it would then be automatically optimized. From the experiments we conducted, setting the radius in our algorithm to a known value would improve the efficiency and fitting accuracy of the algorithm to a certain extent. However, considering the versatility of the algorithm, it was still chosen as an unknown parameter to be solved here.

(4) The fitting accuracy and noise immunity of our algorithm were better than that of the least squares algorithm. It can be seen from Figure 7c that Target 1 and Target 2 had no obvious noise. At this point, the fitting accuracy of the two methods was equivalent. Target 3~4 all contained obvious noises. Especially in the case of obvious outliers in Target 3, our algorithm could still achieve a fitting accuracy of RMSE less than 1 mm, while LS had an obvious large deviation, as shown in Figure 8c. The radius of the actual sphere target used in the experiment was known. From the fitting error of radius, the fitting error of the two algorithms was less than 1 mm when there was no obvious noise influence. However, when there was obvious noise, our algorithm could still be applied stably, while LS was greatly disturbed and had serious deviation, as shown in Figure 8d.

## 4. Discussion

### 4.1. Parameters and Efficiency

The preset radius $R_{\mathrm{set}}$ of the sphere target directly affects the size of the initial space constructed. In TLS work, the sphere target used usually has a fixed geometric radius, in which case $R_{\mathrm{set}}$ could be easily determined. If the radius of the sphere target cannot be determined, a fairly large value could be set. After several iterations of optimization, the range of the radius could be basically determined. According to the experimental results, $R_{\mathrm{set}}$ should be controlled within six times of the actual radius of the sphere target, so that the comparatively accurate radius could be obtained after five iterations. If the value was too large, the fitting accuracy would be low.

When updating the constraint space, the scaling factor α directly determines the contraction speed of the constraint space, and the smaller the value is, the faster the contraction speed is. The experimental results show that α was generally set to about 0.25 to ensure a good fitting effect. At the same time, the setting of α should take into account the point cloud coverage. When the CR was more excellent than 20%, α could be reduced to speed up the search. When the point cloud coverage was less than 20%, the α could be increased to improve the accuracy of the solution.

**Figure 9:**
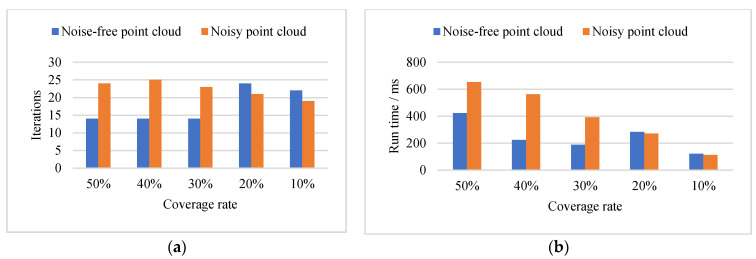
Runtime and iteration optimization times. (**a**) Iterations comparison of two kinds of simulated data. (**b**) Run time comparison of two kinds of simulated data.

The random search times $N_{\mathrm{loop}}$ directly determine the number of samples generated in the finite constraint space, and its value was recommended to be between 500 and 1000. If $N_{\mathrm{loop}}$ was too large, the number of sample points was too large, resulting in low fitting efficiency; if $N_{\mathrm{loop}}$ was too small, the number of samples would be insufficient, resulting in insufficient fitting accuracy. According to the experimental results, when $N_{\mathrm{loop}}$ was set to 1000, all kinds of point clouds could be fitted within 1 s, which was acceptable to us, as shown in Figure 9a. The value of $N_{\mathrm{opt}}$ should be adjusted to that of $N_{\mathrm{loop}}$. If the value of $N_{\mathrm{loop}}$ was relatively small, such as 500, the value of $N_{\mathrm{opt}}$ should be increased. If the $N_{\mathrm{loop}}$ was correspondingly large, such as 1000, it should be possible to reduce the $N_{\mathrm{opt}}$ appropriately. According to the experimental results, when $N_{\mathrm{loop}}$ was set to 1000, an ideal fitting result could generally be achieved within 30 times, as shown in Figure 9b.

The total error threshold $E_{min}$ was the preset condition for the end of the iteration. In the iterative optimization process, if the $E_{total}$ of the current sample was detected to be less than $E_{min}$, the iteration would end in advance, and this sample would be taken as the optimal solution of the sphere target fitting. The value should not be too large, generally less than 0.001. In the algorithm, the check judgment of iterative optimization accuracy was set. If the fitting accuracy of two adjacent iterations was no longer improved, the judgment would be terminated in advance. This judgment was very effective in the fitting process of the noisy point cloud. As shown in Table 2, for the noisy point cloud, the $E_{total}$ at the end of fitting did not reach $E_{min}$, but due to the influence of noise, the precision of $E_{total}$ in the adjacent iterative process did not improve, so the fitting was ended before reaching the preset iteration optimization number $N_{\mathrm{opt}}$.

The time complexity of the proposed algorithm was $O\left(N_{\mathrm{loop}}*N_{\mathrm{opt}}\right)\;$ [52], and the efficiency of the algorithm was directly restricted by $N_{\mathrm{loop}}$ and $N_{\mathrm{opt}}$. It could be seen from the experimental results that several parameters were not absolutely and completely independent, but have some influence on each other. Therefore, the parameter reference value given here was an empirical value, which should be adjusted appropriately according to the actual situation in the practical application of the algorithm.

### 4.2. Robustness and Accuracy

**Figure 10:**
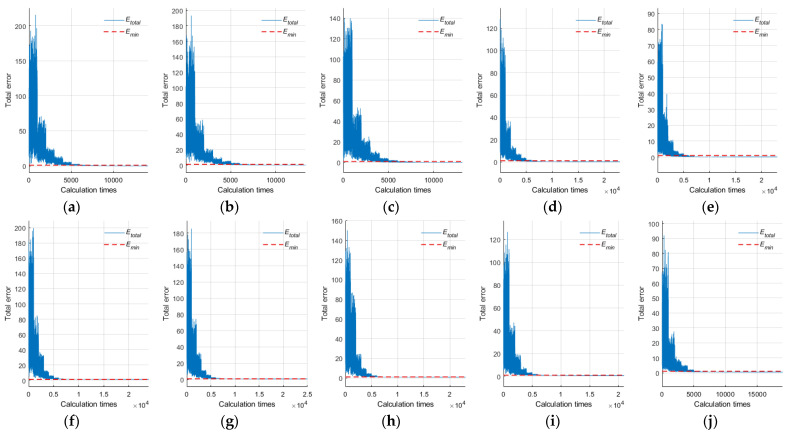
Detailed fitting process of experimental data: (**a**–**e**) were the total error changes during the fitting process of five simulated noise-free point clouds with different coverage rates, (**f**–**j**) were the total error changes during the fitting process of five simulated noisy point clouds with different coverage rates.

Robustness was an important feature of an algorithm. It directly determines its application scope. The proposed algorithm not only makes use of the point cloud data itself but also considers the geometric characteristics of the target sphere, and realizes the target sphere fitting, employing probability and statistic theory. As shown in Figure 10, from the fitting process of 10 simulated sphere targets, regardless of whether there was noise in the sphere target point cloud or not, with the optimization of the constraint space, the total error $E_{total}$ would become smaller and smaller, that is, the fitting accuracy would gradually improve. Generally, it would be basically stable after five iterations, and further optimization would follow.

The sphere target fitting method based on the least-squares completely depends on the target sphere point cloud data, and the noise in the data was easy to cause the ill-conditioned coefficient matrix, which leads to the decrease in fitting accuracy, especially in the case of low coverage rate, it was easy to cause the fitting failure. Although the fitting effect could be improved through error in variables (EIV), observation value weighting, and other strategies, many prior assumptions of such methods were often untenable. In practice, such as equal precision of all measurement points, weight determined by reflection intensity or incident angle, etc. [53,54,55,56,57]. We find the solution from the perspective of probability theory not only relies on the point cloud data but is based on considering the geometric characteristics of the target sphere, through the global optimal parameter estimation to find the best fitting results. This method not only avoids the defects of the least square fitting method but also overcomes the influence of various noises on the fitting accuracy. From the experimental results, when the coverage rate reaches more than 30%, regardless of whether there was noise in the point cloud data, the proposed algorithm could achieve a fitting accuracy of more than 0.01 mm, which was beyond the reach of all current least-square fitting methods. When the coverage rate was less than 20%, the fitting accuracy of the proposed algorithm was reduced to some extent, but it could reach the accuracy of about 1 mm in point cloud fitting with mixed noise. In the verification process, we also conducted fitting tests on point clouds with large noise such as 10 and 15 mm, and found that the proposed algorithm could achieve fitting accuracy of more than 1 mm as long as the coverage rate remained above 20%. This fully proves that the proposed algorithm was practicable and could be used for fitting different sphere target point clouds. 

## 5. Conclusions

Combined with the target sphere’s point cloud and geometric characteristics, a finite random search algorithm was proposed for fast calculation of the center of the target sphere. This algorithm was suitable for all types of sphere target point cloud data and had high fitting accuracy and operation efficiency. According to the experimental results, when the coverage rate reaches more than 20%, regardless of whether the point cloud contains noise, the rapid fitting could be completed within 1 s, and the fitting error was less than 1 mm. When the coverage was less than 20%, the noise-free point cloud could also reach the fitting error of less than 1 mm. When the coverage rate was less than 20%, the fitting error of the noise-free point cloud could also reach within 1mm, while the fitting accuracy of the noisy point cloud was basically within 2 mm, although the accuracy of noisy point cloud decreases relatively.

Future research would focus on the problem of accurate fitting of noise point clouds with low coverage. The noise was removed continuously in the iterative optimization process through spatial clustering or error sorting methods to improve the fitting accuracy further.

## Figures and Tables

**Table 1:** Statistics of fitting results of noise-free point clouds.

Data	Points	CR	Error/mm			RMSE ^1^/mm	Total Error ^2^	Iterations	Runtime/ms
			*dX*	*dY*	*dZ*				
a	3751	50%	−0.0018	0.0006	−0.0010	0.0021	0.0009	14	423.6
b	3267	40%	0.0042	0.0020	0.0064	0.0079	0.0010	14	224.1
c	2783	30%	−0.0035	0.0054	0.0059	0.0087	0.0009	14	188.4
d	2178	20%	−0.0047	0.0009	−0.0400	0.0403	0.0233	24	283.5
e	1573	10%	−0.0001	0.0000	−0.9259	0.9259	0.0853	22	122.0

^1^ RMSE was calculated by Equation (6). ^2^ Total error at the end of the fitting.

**Table 2:** Statistics of fitting results of noisy point clouds.

Data	Points	CR	Error/mm			RMSE ^1^/mm	Total Error ^2^	Iterations	Runtime/ms
			*dX*	*dY*	*dZ*				
a	3751	50%	0.0000	0.0000	0.0000	0.0000	1.1020	24	654.1
b	3267	40%	0.0000	0.0000	0.0002	0.0002	0.9799	25	563.1
c	2783	30%	0.0000	0.0000	0.0000	0.0000	0.8362	23	392.0
d	2178	20%	0.0000	0.0003	0.2612	0.2612	0.6271	21	271.8
e	1573	10%	0.2437	−0.1620	−1.9100	1.9323	0.5343	19	112.8

^1^ RMSE was calculated by Equation (6), ^2^ total error at the end of the fitting.

**Table 3:** Comparison of fitting results of five point clouds.

Target	Our Algorithm/mm					Least Squares/mm				
	*dX*	*dY*	*dZ*	*dR*	*RMSE*	*dX*	*dY*	*dZ*	*dR*	*RMSE*
1	0.1115	0.0285	0.0666	0.0832	0.1330	0.3854	0.0241	0.1299	0.3480	0.4074
2	0.4377	0.1325	0.1392	0.3615	0.4780	0.3777	0.1454	0.1224	0.3120	0.4228
3	0.3654	0.4141	0.1136	0.4644	0.5638	4.8472	3.5357	1.5258	5.0020	6.1906
4	0.2010	0.5261	0.0027	0.8077	0.5632	0.3924	2.9402	0.6130	2.5100	3.0289
5	0.4966	0.7367	0.3502	0.5013	0.9549	1.0523	1.3143	1.2507	1.1236	2.0973

## Data Availability

The data presented in this study are available on request from the corresponding author.
